# An Acquisition Method of Agricultural Equipment Roll Angle Based on Multi-Source Information Fusion

**DOI:** 10.3390/s20072082

**Published:** 2020-04-07

**Authors:** Yang Li, Honglei Jia, Jiangtao Qi, Huibin Sun, Xinliang Tian, Huili Liu, Xuhui Fan

**Affiliations:** 1 Key Laboratory of Bionic Engineering, Ministry of Education, Jilin University, Changchun 130022, China; l_yang13@mails.jlu.edu.cn (Y.L.); jiahl@vip.163.com (H.J.); 16688206566@163.com (H.S.); t18094802479@163.com (X.T.); liuhuili@163.com (H.L.); 2 College of Biological and Agricultural Engineering, Jilin University, Changchun 130022, China; 3 Graduate School of Agriculture, Kyoto University, Kyoto 6068502, Japan; 4 Agricultural Machinery Research Institute of Jilin Province, Changchun 130021, China; cchkbjomfan@163.com

**Keywords:** agricultural equipment, roll angle, Kalman filter, sensor data fusion

## Abstract

Accurately obtaining roll angles is one of the key technologies to improve the positioning accuracy and operation quality of agricultural equipment. Given the demand for the acquisition of agricultural equipment roll angles, a roll angle monitoring model based on Kalman filtering and multi-source information fusion was established by using the MTi-300 AHRS inertial sensor (INS) and XW-GI 5630 BeiDou Navigation Satellite System (BDS), which were installed on agricultural equipment. Data of the INS and BDS were fused by MATLAB; then, Kalman filter was used to optimize the data, and the state equation and measurement equation of the integrated system were established. Then, an integrated monitoring terminal man–machine interactive interface was designed on MATLAB GUI, and a roll angle monitoring system based on the INS and BDS was designed and applied into field experiments. The mean absolute error of the integrated monitoring system based on multi-source information fusion during field experiments was 0.72°, which was smaller compared with the mean absolute errors of roll angle monitored by the INS and BDS independently (0.78° and 0.75°, respectively). Thus, the roll angle integrated model improves monitoring precision and underlies future research on navigation and independent operation of agricultural equipment.

## 1. Introduction

Intelligent agricultural machinery equipment is one key technique of modern agriculture, and its intellectualization is increasingly higher and its working width is gradually broadened. During field operations, the attitude angles of wide-amplitude agricultural machine, especially the roll angle, affect the tillage depth and compressing strength of machine and thereby impact the tillage quality [1,2]. When an agricultural machine is turning or driving on a curved line in farmlands, the accurate roll angle of the implement can provide support for the control of automatic navigation agricultural vehicles [3], and it will prewarn the possible roll before climbing or descending, thereby effectively decreasing the loss of life and properties [4].

The roll angle of agricultural machine can be measured in multiple ways [5,6,7,8,9], and the commonly used method is based on inertial sensors. Inertial sensors generally include accelerometers and gyroscopes [10,11,12,13] or inclination sensors [14,15]. For instance, an external acceleration inclination angle Kalman filter algorithm was proposed for agricultural machinery [1]. Two SensComp 600 ultrasonic sensors were used to measure the reference level heights at two sides of flat shovels respectively, and the inclining angles of flat shovels were determined according to geometrical relations [13]. 

Regarding the limitations of using only one single sensor, researchers have tried to combine multiple sensors to improve the perceived precision of attitude angles. Zheng et al. established a multi-sensor fusion algorithm and an attitude decision model based on gyroscopes and accelerometers and applied them into the real-time analysis and detection of carrier attitudes [16]. The information of micro-electro mechanical system (MEMS) gyroscopes and accelerometers was fused for real-time inclination measurements of scrapers [17,18]. Vargas-Meléndez et al. presented a novel estimator based on sensor fusion, which combined the neural network with a Kalman filter in order to estimate the vehicle roll angle [19]. Garcia et al. used MEMS sensors combined with data fusion algorithms to estimate sideslip or inclination angles [20,21]. Based on the multi-sensor system integrating a gyroscope, accelerometer, and magnetometer, Zhang et al. designed a novel dual-linear Kalman filter [22]. Wu et al. developed a new method for attitude estimation by fusing measurements from both the gyroscope and the accelerometers [23]. Kamal et al. designed complementary and Kalman filters for the attitude estimation of a vehicle based on a low-cost inertial measurement unit [24]. Khot et al. used a discrete Kalman filter to integrate the attitude angles, which were obtained from a digital elevation model and a terrain compensation module sensor, to improve the roll and pitch angle estimates of a self-propelled sprayer [25].

In practical applications, the measurement error of an inertial navigation sensor will increase over time. The global satellite positioning system has high navigation accuracy, and the position, velocity, and attitude information of the solution will not cause errors due to time accumulation. So, the calculated navigation information can be used to correct the drift of INS data. Therefore, this study was targeted at developing a roll angle monitoring algorithm based on the INS and BDS. In brief, a roll angle acquisition model was built based on the INS and BDS by using multi-source information fusion, and a roll angle integrated system was designed and applied to field experiments, which provides a new method for improving the roll angle acquisition accuracy.

## 2. Materials and Methods 

### 2.1. Definition of Three-Dimensional Attitude Angles 

**Figure 1:**
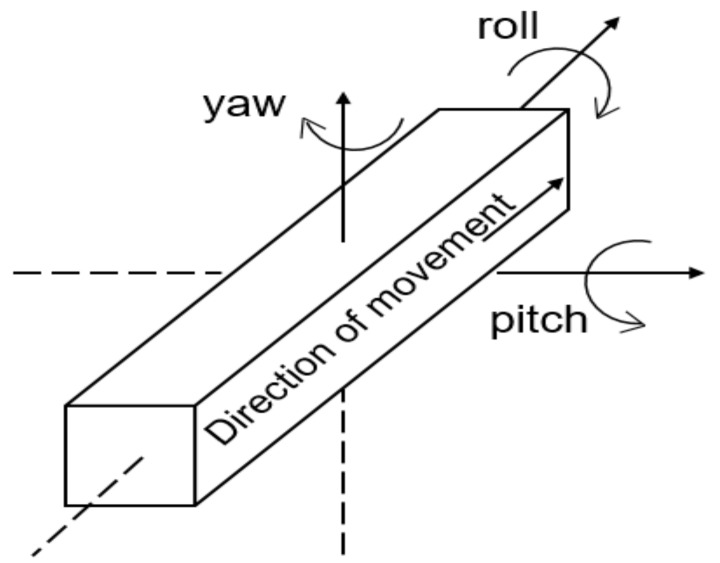
Definition of three-dimensional attitude angles.

Let the 3D attitude angles of an object be the roll angle φ, pitch angle θ, and yaw angle ψ, which are the three angles of a carrier on a local horizontal coordinate system (Figure 1) [26]. 

Specifically, the roll angle is the rotation angle when the object revolves around the direction of movement, and it varies within [−180°, 180°]. The yaw angle is the rotation angle around the vertical line at the motion direction (namely, the rotation angle of the moving object relative to the north direction on the geographic coordinate system (GCS)), and it varies within [0°, 360°]. The pitch angle is the rotation angle around the vertical axis, and it varies within [−90°, 90°].

### 2.2. INS and Performances 

**Figure 2:**
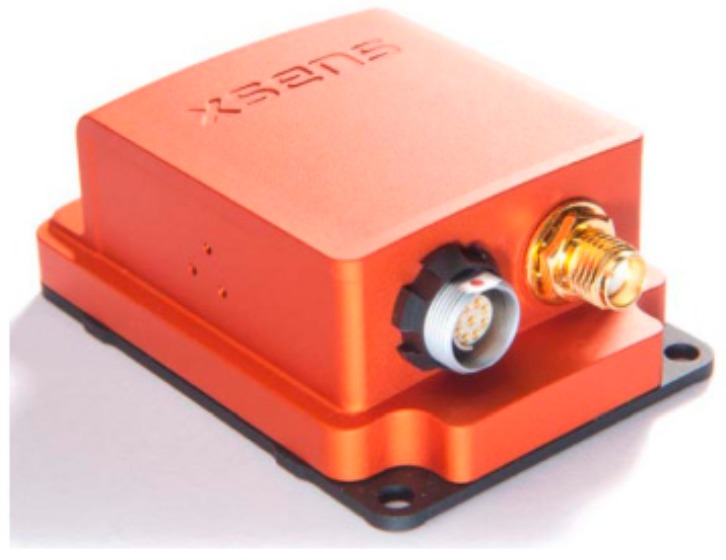
The MTi-300 AHRS sensor.

The MTi-300 AHRS INS used in this study (Figure 2) belongs to MEMS sensors and mainly consists of a 3-shaft silicon micro-mechanical accelerometer, a 3-shaft silicon micro-mechanical gyroscope, and a 3-shaft magnetometer. Inside the INS, the data were fused at low power consumption. Through a USB serial communication bus interface, the 3-shaft acceleration, 3-shaft angular velocity, 3-shaft magnetic field intensity, and attitude were output in real time [27]. 

### 2.3. BDS and Performances 

**Figure 3:**
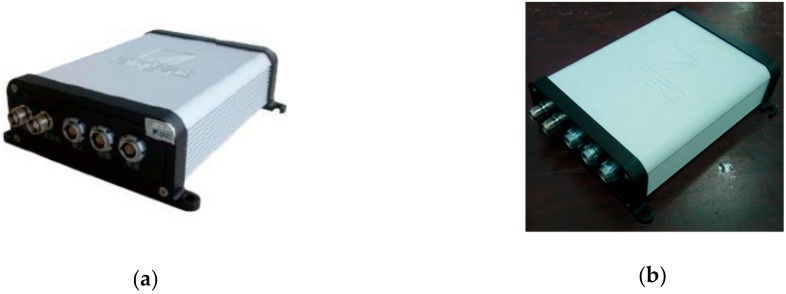
BeiDou Navigation Satellite System (BDS) used in this study: (**a**) The XW-GI 5630 mobile station; (**b**) The XW-GNSS 1060 base station.

BDS consists of a space segment, a ground segment, and a user segment. The ground segment involves main control stations, injection stations, monitoring stations, and other ground stations; the user segment includes the Beidou user terminals and the terminals that are compatible with other satellite navigation systems [28]. The BDS (Beijing Starneto Technology Co. Ltd., Beijing, China) used in this study had two receivers: the mobile station XW-GI 5630 and the base station XW-GNSS 1060 (as shown in Figure 3). The system can output the position, speed, attitude, and other information of the carrier in real time.

### 2.4. Fused Monitoring of the INS and BDS 

**Figure 4:**
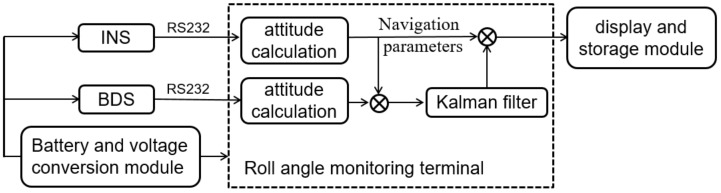
Structural diagram of the sensor fusion system.

INS cannot independently work for a long time because its errors gradually rise with time. The position information, velocity information, and attitude information of the BDS do not drift with time, so its navigation information can be used to correct the drift of the INS data. However, because the position of the agricultural equipment is constantly changing, when the satellite signal is blocked by tall buildings or trees, or it is interfered by radio waves, the receiver may not capture the satellite signal, which will invalidate the measurement results. To fully utilize the advantages of these two techniques, the INS and BDS were integrated by using the sensor data fusion technique, and a rolling angle monitoring platform for agricultural equipment was established. The sensor fusion system was structurally illustrated in Figure 4 and it mainly consisted of the INS, the BDS, and the fusion monitoring terminal.

The sensor fusion system worked as follows: the BDS receiver outputs the velocity and position of the carrier, which were transferred by an RS232 serial port into the fusion monitoring terminal, so the BDS can compensate for the accumulative error of the INS. The data of the INS were first read and resolved by the monitoring terminal, and then the attitude angle, position, and velocity of the carrier were calculated and subtracted with the position and velocity of the BDS, forming the results of measured data. After that, the errors of the INS were estimated by the Kalman filter. 

When the BDS signals were invalid, the system will switch to the INS sole working mode; when the BDS signals were valid, the BDS can correct the carrier attitude. 

### 2.5. Implementation of the INS and BDS Integrated Algorithm 

This paper adopts the integrated navigation scheme on software. In brief, the measured data from the BDS and from the INS were sent to the fusion monitoring terminal, where the spatiotemporal synchronization of data was first done by writing a program with GUI (graphical user interface) on MATLAB and then Kalman filtering was finished on MATLAB to achieve fusion and correction. 

The data of the INS and BDS were processed by using a centralized Kalman filter [29]. In brief, the observed data from the BDS and INS were processed by the optimal error estimation of Kalman filtering, so as to correct the original system and produce high-precision attitude data. 

In the meantime, the INS independently will work for short time when the BDS signals were invalid, since the INS was corrected by the Kalman filter, the output time accumulated error of the INS was not too large and the INS can fully independently monitor attitude until the BDS obtained satellite signals again. 

(1) Establishment of state equation. 

The state variables of the system are:

(1) $$X=\left[\delta L\;\delta \lambda \;\delta v_{E\;}\delta v_{N}\;\varphi_{E\;}\varphi_{N\;}\varphi_{U\;}\varepsilon_{rx\;}\varepsilon_{ry\;}\varepsilon_{rz\;}\nabla_{x}\;\nabla_{y}\;\nabla_{z}\right]$$

where δL, δλ are errors of latitude and longitude respectively; $\;\delta v_{E,}\delta v_{N}$ are errors of eastward and northward velocity respectively; $\varphi_{E},\;\varphi_{N},\;\varphi_{U}$ are errors of eastward, northward and upward attitude angle respectively; $\varepsilon_{rx},\varepsilon_{ry},\varepsilon_{rz\;}$ are the errors of gyro random drift respectively; $\nabla_{x},\;\nabla_{y},\;\nabla_{z}\;$ are the zero bias of the accelerometer respectively.

Its state equation is:

(2) $$\hat{X}=FX+G\omega$$

where ***F*** is the transfer matrix of state ***X***; the noise of the system is $\omega =\left[\omega_{gx}\;\omega_{gy}\;\omega_{gz}\;\omega_{bx}\;\omega_{by}\;\omega_{bz}\;\omega_{ax}\;\omega_{ay}\;\omega_{az}\right]$, where $\omega_{gx},\;\omega_{gy},\;\omega_{gz}$ are the white noise of gyro drift respectively, $\omega_{bx},\;\omega_{by},\;\omega_{bz}$ are the Markov white noise of random drift respectively; $\omega_{ax},\omega_{ay},\;\omega_{az}$ are the zero-bias 1st-order Markov white noise of the angular velocity meter respectively.

Where $G=\left[\begin{array}{ccc} 0_{3\times 3} & 0_{3\times 3} & 0_{3\times 3}\\ 0_{1\times 3} & 0_{1\times 3} & 0_{1\times 3}\\ I_{3\times 3} & 0_{3\times 3} & 0_{3\times 3}\\ 0_{3\times 3} & I_{3\times 3} & 0_{3\times 3}\\ 0_{3\times 3} & 0_{3\times 3} & I_{3\times 3} \end{array}\right]$.

The nonzero element of ***F*** is:

$$\begin{array}{l} F_{1,4}=\frac{1}{R+h},\;F_{2,1}=\frac{v_{e}}{cosL\left(R+h\right)\;}tanL,\;F_{2,3}=\frac{1}{cosL\left(R+h\right)},\;\\ F_{3,1}=2\omega_{ie}cosLv_{n}+\frac{v_{e}v_{n}}{cos^{2}L\left(R+h\right)}+2\omega_{ie}sinLv_{u},\;F_{3,3}=\frac{v_{n}}{R+h}tanL-\frac{v_{u}}{R+h},\\ F_{3,4}=2\omega_{ie}sinL+\frac{v_{e}}{cosL\left(R+h\right)},\;F_{3,6}=-f_{u},\;F_{3,7}=f_{n},\;F_{3,11}=1,\;\\ F_{4,1}=-\left(2\omega_{ie}cosLv_{n}+\frac{v_{e}^{2}}{cos^{2}L\left(R+h\right)}\right),\;F_{4,4}=-\frac{v_{u}}{R+h},\;F_{4,3}=-\left(2\omega_{ie}sinL+\frac{v_{e}}{R+h}tanL\right),\;\\ F_{4,7}=-f_{e},\;F_{4,5}=\varphi_{u},\;F_{4,12}=1,\;F_{5,4}=-\frac{1}{R+h},\;F_{5,6}=\omega_{ie}sinL+\frac{v_{e}}{R+h}tanL,\;\\ F_{5,7}=-\left(2\omega_{ie}cosL+\frac{v_{e}}{R+h}\right),\;F_{5,8}=1,\;F_{6,1}=-\omega_{ie}sinL,\;F_{6,3}=\frac{1}{R+h}\\ F_{6,5}=-\left(\omega_{ie}sinL+\frac{v_{e}}{R+h}tanL\right),\;F_{6,7}=-\frac{v_{n}}{R+h},\;F_{6,9}=1,\;F_{7,1}=\omega_{ie}cosL+\frac{v_{e}}{cos^{2}L\left(R+h\right)},\;\\ F_{7,3}=\frac{tanL}{R+h},\;F_{7,5}=\omega_{ie}cosL+\frac{v_{e}}{R+h},\;F_{7,6}=\frac{v_{n}}{R+h},\;F_{7,10}=1,\;F_{7,10}=-\frac{1}{\tau_{g}},\;F_{10,10}=-\frac{1}{\tau_{g}}\\ F_{9,9}=-\frac{1}{\tau_{g}} \end{array}$$

where *R* is the Earth’s radius (637,000 m). 

(2) Establishment of measurement equation. 

The measured data of the system include the measurement errors of position and velocity. The measured position information of the MTi-300 AHRS sensor can be expressed as the sum of the real data and corresponding errors under GCS:

(3) $$\left[\begin{array}{c} L_{MTi}\\ \lambda_{MTi}\\ h_{MTi} \end{array}\right]=\left[\begin{array}{c} L_{t}+\delta L\\ \lambda_{t}+\delta \lambda \\ h_{t}+\delta h \end{array}\right]$$

where ***L_t_,*** ***λ_t_, h_t_*** are the real position respectively; ***δ_L_,*** ***δ_λ_,*** ***δ_h_*** and ***L_MTi_,*** ***λ_MTi_, h_MTi_*** are the position errors and measured values of MTi-300 AHRS at the eastward, northward, and upward direction, respectively.

The measured information of position from the satellite receiver can be expressed as the difference between the real data and corresponding errors under GCS:

(4) $$\left[\begin{array}{c} L_{BDS}\\ \lambda_{BDS}\\ h_{BDS} \end{array}\right]=\left[\begin{array}{c} L_{t}-\frac{N_{n}}{R}\\ \lambda_{t}-\frac{N_{e}}{RcosL}\\ h_{t}-N_{u} \end{array}\right]$$

where ***L_t_,*** ***λ_t_****,* and ***h_t_***, are the real position respectively; ***N_n_, N_e_, N_u_*** and ***L_BDS_,*** ***λ_BDS_, h_BDS_*** are the position errors and measured values of the satellite receiver at the eastward, northward, and upward directions, respectively; R is the Earth’s radius; and L is the tested precision.

The velocity-measured information of the INS can be denoted as the sum of real data and corresponding velocity errors under GCS:

(5) $$\left[\begin{array}{c} v_{eMTi}\\ v_{nMTi}\\ v_{uMTi} \end{array}\right]=\left[\begin{array}{c} v_{e}+\delta v_{e}\\ v_{n}+\delta v_{n}\\ v_{u}+\delta v_{u} \end{array}\right]$$

where ***v_e_, v_n_****,* and ***v_u_*** are the real velocity, respectively; while ***δ_ve_,*** ***δ_vn_,*** ***δ_vu_*** and ***v_eMTI_, v_nMTI_, v_uMTI_*** are the velocity errors and measured values at the eastward, northward, and upward directions in the geographic coordinate system, respectively.

The velocity measured information of satellites can also be denoted as the sum of real data and the resolved velocity errors under GCS:

(6) $$\left[\begin{array}{c} v_{BDSx}\\ v_{BDSy}\\ v_{BDSz} \end{array}\right]=\left[\begin{array}{c} v_{x}-M_{x}\\ v_{y}-M_{y}\\ v_{z}-M_{z} \end{array}\right]$$

where ***v_x,_ v_y_, v_z_*** are the real velocity, respectively; ***M_x_, M_y_, M_z_*** and ***v_BDSx_, v_BDSy_, v_BDSz_*** are the velocity errors and measured values of the receiver at the eastward, northward, and upward directions, respectively. Hence, the measurement equation of the combined position and speed of the integrated system is:

(7) $$Z\left(t\right)=\left[\begin{array}{c} L_{MTi}-L_{BDS}\\ \lambda_{MTi}-\lambda_{BDS}\\ h_{MTi}-h_{BDS}\\ v_{eMTi}-v_{eBDS}\\ v_{nMTi}-v_{nBDS}\\ v_{uMTi}-v_{uBDS} \end{array}\right]=H\left(t\right)X\left(t\right)+V\left(t\right)$$

where the observation matrix is $H\left(t\right)=\left[\begin{array}{ccccccccccccccc} R & 0 & 0 & 0 & 0 & 0 & 0 & 0 & 0 & 0 & 0 & 0 & 0 & 0 & 0\\ 0 & RcosL & 0 & 0 & 0 & 0 & 0 & 0 & 0 & 0 & 0 & 0 & 0 & 0 & 0\\ 0 & 0 & 1 & 0 & 0 & 0 & 0 & 0 & 0 & 0 & 0 & 0 & 0 & 0 & 0\\ 0 & 0 & 0 & 1 & 0 & 0 & 0 & 0 & 0 & 0 & 0 & 0 & 0 & 0 & 0\\ 0 & 0 & 0 & 0 & 1 & 0 & 0 & 0 & 0 & 0 & 0 & 0 & 0 & 0 & 0\\ 0 & 0 & 0 & 0 & 0 & 1 & 0 & 0 & 0 & 0 & 0 & 0 & 0 & 0 & 0 \end{array}\right]$.

### 2.6. Design of Fusion Monitoring Terminal 

**Figure 5:**
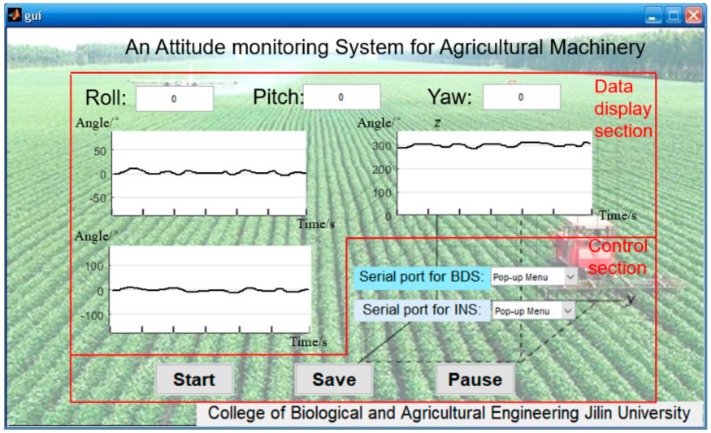
Man–machine interaction interface.

Data of the INS and BDS were fused through the program compiled on MATLAB GUI, and then an integrated monitoring system was designed (Figure 5), which was responsible for the acquisition, display, and storage of test data in real time. The software interface consists of two parts: a data display section and a control section. The control section is responsible for system operation, data storage, and pause, and the data display shows the changes of the roll angle. 

**Figure 6:**
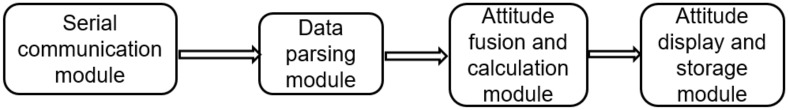
Overall structure of the software system.

A modular design was finished on MATLAB, including the serial port communication module, data parsing module, attitude fusion and calculation module, and attitude display and storage module. The overall structure of the system is shown in Figure 6. 

In the serial communication module, the communication mode of both systems was the serial port asynchronous communication, and the software firstly configured the serial port. Prior to asynchronous communication, the communication protocol was set, and the serial port communication parameters of the INS and BDS were the same: a baud rate of 115,200 bps, data bit of 8, stop bit of 1, and no parity bit. The output data of the INS conforms to the standard format of NMEA (National Marine Electronics Association) protocol, such as $HCHDM, $HCHDG, and $PSONCMS and so on. The quaternion, acceleration, and angular velocity were extracted by selecting $PSONCMS so as to determine the velocity, position, and attitude. The output data of BDS also conforms to the standard format of NMEA protocol. Here, $GPFPD was selected to acquire the velocity, position, and attitude, and the data structure was $GPFPD, GPSWeek, GPSTime, Heading, Pitch, Roll, Lattitude, Longitude, Altitude, Ve, Vn, Vu, Baseline, NSV1, NSV2, and Status *cs<CR><LF>, where “$” is the beginning mark of data, and “,” is the separation field mark. 

In the data parsing module, the required information is obtained by analyzing and extracting the collected data. In the MATLAB program, the timer function is used to periodically read the data in the serial buffer. When “$” is read, the data in the corresponding position is parsed and extracted. If it is not “$”, continue to read the next character until the “$” is read. The data were saved in the format of texts, and the program stored the data in the order of time, pitch angle, roll angle, and yaw angle. The man–machine interface in the attitude display module was designed by using MATLAB GUI. The GUI data update display module mainly consists of the data display part and the curve part, and the update frequency is once per second, which is completed by the timer.

## 3. Experiments and Analysis 

### 3.1. Roll Angle Monitoring Test Based on the INS 

#### 3.1.1. Test Conditions and Methods 

In order to test the accuracy of the INS measurement system, experiments were carried out at Jilin University during the operations of plowing and sowing in spring. During the test, the INS is fixed on the top of the tractor, and the tractor battery provided power to the equipment. The monitoring terminal is used to receive and record the output data of the INS in the cab.

**Figure 7:**
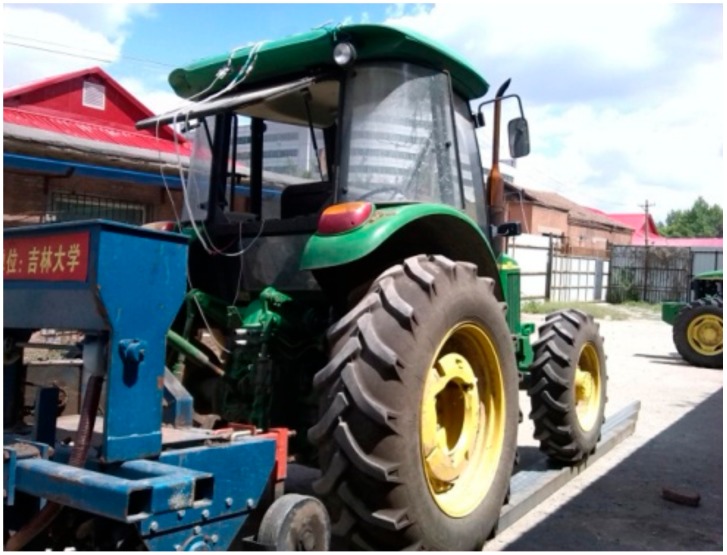
Attitude dynamic measurement by the MTi–300 AHRS sensor.

Firstly, a 10-m long trail with a certain height is set on a flat surface. When the tractor was run at a constant speed of 1 m/s, the tire on one side passed through the trail. When the tractor passed through the trail, the monitoring terminal was used to record and save the INS data, and an electronic level bar was used to record the roll angle at this time. The testing site was illustrated in Figure 7. When the height of the high-level place was adjusted, the tractor passing through the high-level place formed different roll angles. According the results of the communique of the second national land survey data main achievements [30], the area of land that has a titled angle less than 2° is 57.1% in China. Hence, the height of the high-level place was adjusted to make the roll angle fall within –4° and 4°. During this process, data were recorded by an electronic level bar 10 times and used as reference, and the sampling frequency of the INS was 10 Hz.

#### 3.1.2. Results and Analysis 

**Figure 8:**
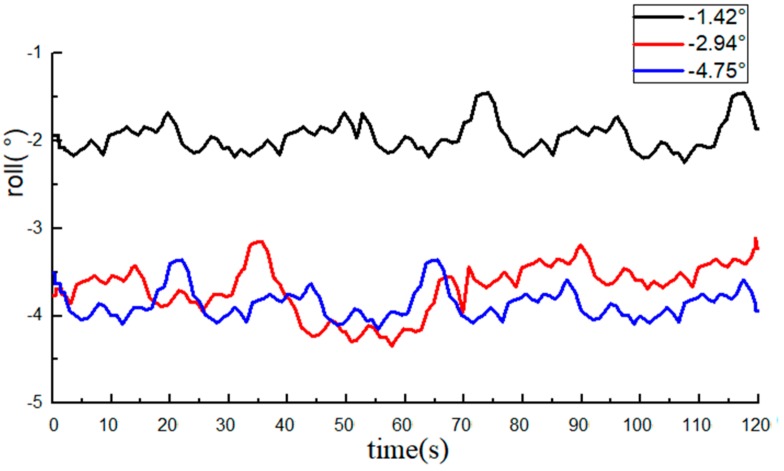
Data from the inertial sensor (INS, roll angle data are all negative).

**Figure 9:**
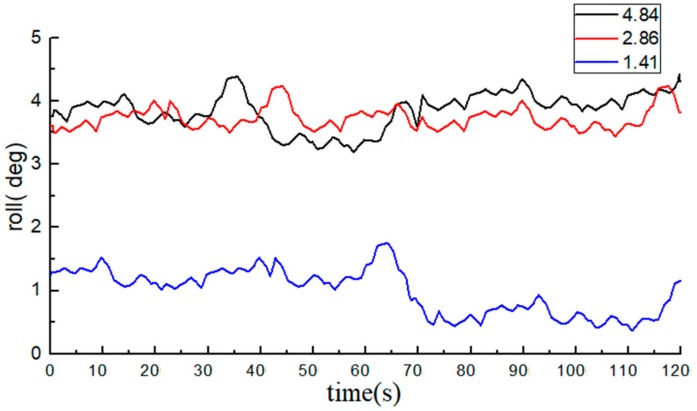
Data from the INS (roll angle data are all positive).

The roll angle data recorded by the INS are shown in Figure 8 and Figure 9. The average value of the 10 measurements by an electronic level bar was used as the reference and compared with the data from the INS. The statistical data are listed in Table 1. 

The measurements of the roll angle imply that during the dynamic straight-line driving of the tractor, the least absolute error of the roll angle measurement by the INS is 0.49°, the maximum absolute error is 0.98°, and the average absolute error is 0.78°, indicating that the INS can effectively monitor the roll angle of tractors. 

### 3.2. Roll Angle Monitoring Based on BDS 

#### 3.2.1. Test Conditions and Methods 

To validate the roll angle measurement accuracy of the BDS, experiments were carried out at Jilin University during the operations of plowing and sowing in spring. The experimental plot had a broad space with few obstacles, and the testing day was sunny. 

The BDS mobile station was connected with the monitoring terminal, and the test methods were the same as those shown in Section 3.1. In brief, the roll angle was monitored by the BDS and simultaneously measured by an electronic level bar.

**Figure 10:**
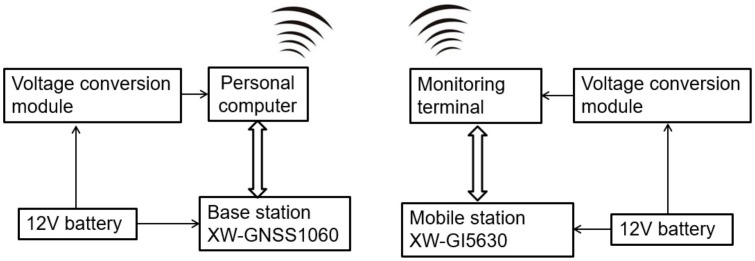
Sketch maps of connection between the base station and mobile station of the BDS.

**Figure 11:**
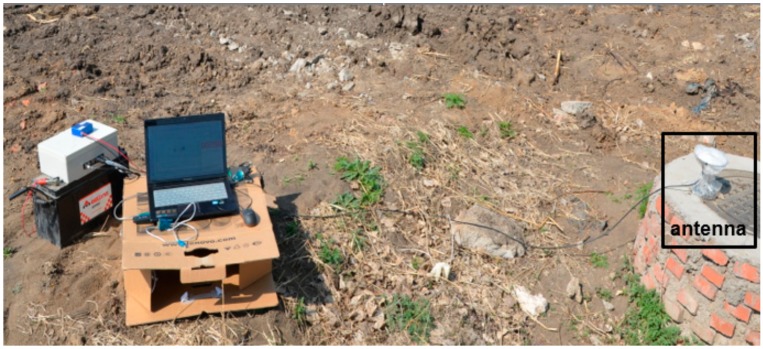
Base station of the BDS.

In the BDS, the XW-GNSS 1060 base station offered differential signals to the XW-GI 5630 mobile station, so the differential method was used to the positioning and attitude measurement. The connection between the two stations was illustrated in Figure 10. Prior to the experiments, the base station connected, at a fixed position, to a laptop via a serial port line. After the base station was positioned successfully, the data transmitted from the serial port was delivered by a personal computer to the Internet. The mobile station was connected via a serial port cable to the monitoring terminal, where it acquired the data (delivered by the base station) from the Internet so as to correct its own data. The data from the XW-GI 5630 mobile station were recorded on the monitoring terminal. The data updating frequency was 10 Hz, and the base station is illustrated in Figure 11. 

#### 3.2.2. Results and Analysis 

**Figure 12:**
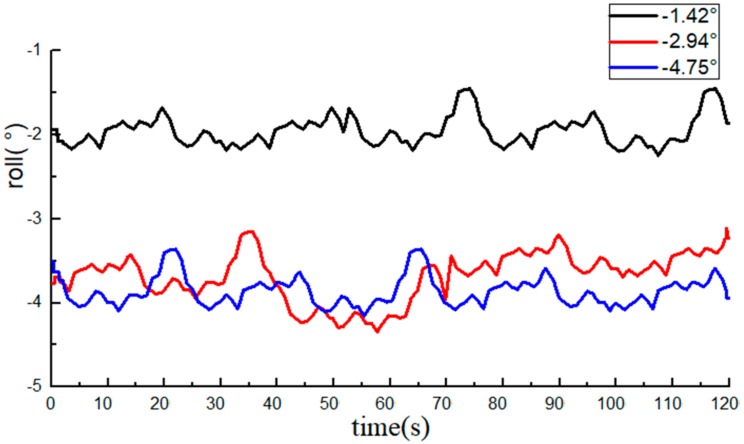
Data from the BDS (roll angle data are all negative).

**Figure 13:**
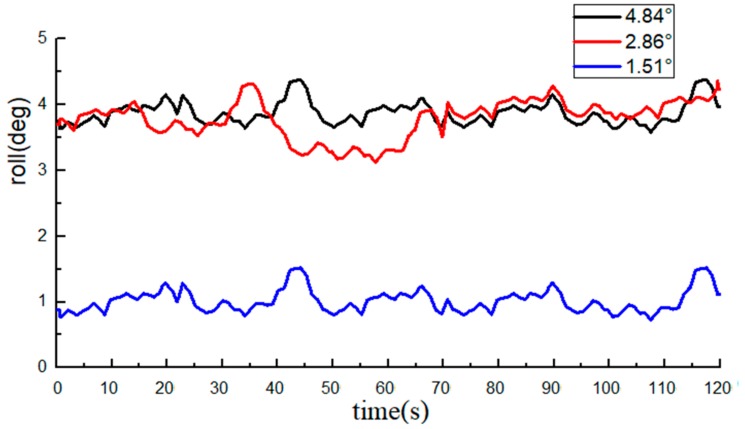
Data from the BDS (roll angle data are all positive).

With the average of 10 measurements of the electronic level bar as the reference, the BDS collected 1200 data at each angle. The data of the roll angle at each angle are shown in Figure 12 and Figure 13. 

The average value of measurements from the high-precision electronic level bar was used as the reference and compared with the post-processing roll angle data from the BDS. The statistical data are listed in Table 2. 

The measurements of the roll angle imply that during the dynamic straight-line driving of the tractor, the least absolute error of the roll angle based on the BDS is 0.49°, the maximum absolute error is 0.95°, and the average absolute error is 0.75°, indicating that the BDS can effectively predict the roll angle of tractors. From the table, we can see that the errors of individual data were very large (e.g., the roll angle was at the standard value of 4.84°). The reason was attributed to the errors due to tractor vibration, and if the data of the BDS at this moment decreased or were lost, the monitoring precision will also be lowered. 

Comparison of data from the BDS and the INS implied that the measurement errors of the BDS and the INS were both around 0.9°, but the precision of the BDS was slightly higher than that of the INS. 

### 3.3. Roll Angle Precision Monitoring Based on the Integrated System 

#### 3.3.1. Experimental Conditions 

Roll angle monitoring experiments were conducted on the farmlands with moderate varying slopes in the experimental plot of Jilin University. The experimental plot had a broad space, and the testing day was sunny. 

The experimental instruments included a 1GZL-3 tillage machine, a John Deere 904 tractor, and the roll angle integrated monitoring system. 

#### 3.3.2. Experimental Methods 

The velocity, position, acceleration, and angular velocity were detected and analyzed. Every period of the experiments was 120 s, the sampling frequency of the INS was 10 Hz, and the sampling frequency of the BDS was 10 Hz. 

**Figure 14:**
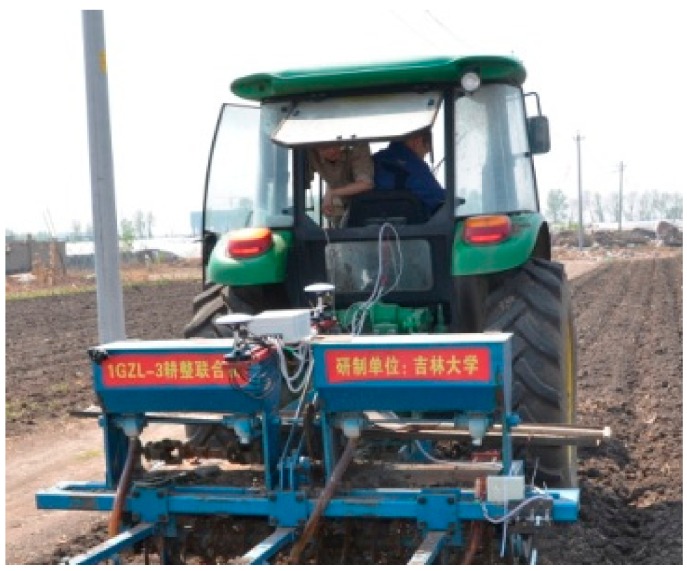
Field dynamic experiments.

During the experiments, the facilities were installed as shown in Figure 14. The heading direction of the iron frames was fixed at the middle of two fertilizer bins. The front and back antennas of the BDS were installed, one in front and one at back, onto the iron frames along the heading direction of the facilities. The magnetic receivers at the bottom of antennas can firmly attach the antennas onto the iron frames. Then, the BDS receiver and the INS were installed in the middle of the iron frames. The tractor hitches the implement to pass at a constant speed of 1 m/s on the ground with a certain slope, and the data from the integrated system were recorded and saved by the monitoring terminal. The real values of the roll angle were recorded by an electronic level bar.

Each group of experiments lasted about 120 s. During the combined monitoring, the testing process was separated, through artificial shielding of satellite signals, into satellite working intervals (0–50 s, 70–120 s) and the satellite missing interval (50–70 s). 

#### 3.3.3. Results and Analysis 

**Figure 15:**
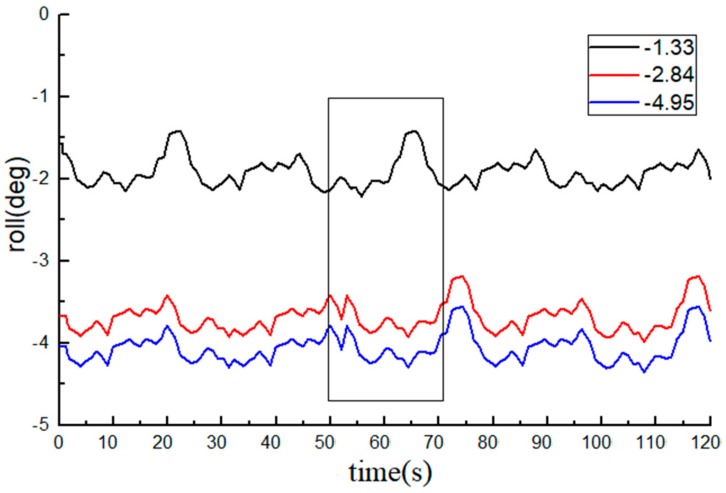
Dynamic data from the integrated system (roll angle data are all negative).

**Figure 16:**
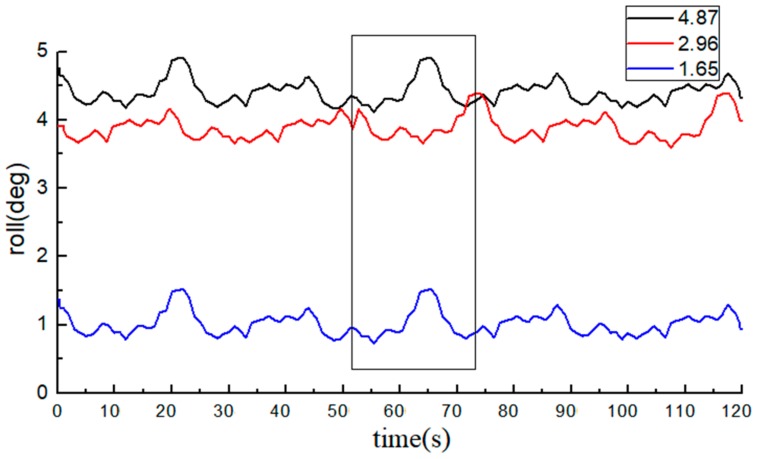
Dynamic data from the integrated system at different measuring angles (roll angle data are all positive).

Figure 15 and Figure 16 show the roll angles recorded by the integrated system during field experiments. The average value of measurements from the DEVON—an electronic level bar—was used as the reference and compared with the roll angle data from the INS. The statistical data are listed in Table 3. 

Measurements show that during the straight-line driving of the tractor in farmlands, the least absolute error of roll angle from the INS and BDS integrated system is 0.46°, the maximum absolute error is 0.93°, and the average absolute error is 0.73°. The above roll angle curves indicate that the integrated system can well measure the roll angle of the agricultural machine during the whole testing period, and even when the satellite signals were invalid (from 50 to 70 s), it still worked well. 

### 3.4. Discussion 

Prior to the use of the multi-source information fusion algorithm, the absolute mean errors were 0.78° and 0.75° respectively when the roll angle was measured by the INS and BDS independently. After processing by the Kalman filtering fusion algorithm, the average relative error of the integrated system was 0.73°, indicating that the fusion algorithm can effectively decrease the observational errors. 

The feasibility of the integrated system was also validated by field experiments, and it can still accurately measure the roll angle of the tractor when the satellite data were temporally invalid (from 50 to 70 s). 

## 4. Conclusions 

(1) A roll angle monitoring model based on multi-source information fusion was built, and a roll angle integrated monitoring system was designed and applied into field experiments, which underlie the research on the navigation and independent driving of the agricultural equipment. 

(2) The INS/BDS integrated system was adopted according to the software. In brief, the data of the roll angle from the INS and BDS were sent to the fusion monitoring terminal, where Kalman filtering and data fusion were accomplished in MATLAB. A monitoring system was designed based on MATLAB GUI and used for the acquisition, display, and storage of real-time experimental data. 

(3) Field experiments based on the INS, BDS, and their integrated system were conducted to monitor a roll angle. With the INS, the least absolute error of the roll angle is 0.49°, the maximum absolute error is 0.98°, and the average absolute error is 0.78°. With the BDS, the least absolute error of the roll angle is 0.49°, the maximum absolute error is 0.95°, and the average absolute error is 0.75°. With the integrated system, the least absolute error of the roll angle is 0.46°, the maximum absolute error is 0.93°, and the average absolute error is 0.73°. Comparisons indicate that the integrated monitoring precision based on multi-source information fusion is higher, which underlies the research on monitoring the roll angle of agricultural equipment.

## Figures and Tables

**Table 1:** Data from the INS.

Reference/°	Measured Value of the INS/°	Standard Deviation of the INS/°	Absolute Error/°	Mean Absolute Error/°
−1.43	−1.92	0.41	0.49	0.78
−2.94	−3.76	0.42	0.82	
−4.75	−3.78	0.38	0.97	
4.84	3.86	0.29	0.98	
2.86	3.73	0.47	0.87	
1.51	0.99	0.45	0.52	

**Table 2:** Measurement of roll angle from the BDS.

Reference/°	Measured Value of the BDS/°	Standard Deviation of the BDS/°	Absolute Error/°	Mean Absolute Error/°
−1.43	−1.95	0.21	0.52	0.75
−2.94	−3.68	0.29	0.74	
−4.75	−3.86	0.31	0.89	
4.84	3.89	0.29	0.95	
2.86	3.79	0.35	0.93	
1.51	1.02	0.18	0.49	

**Table 3:** Dynamic experiment results of the integrated system.

Measured Value of the Ruler/°	Measured Value of the System/°	Standard Deviation of the System/°	Absolute Error/°	Mean Absolute Error/°
−1.33	−1.92	0.31	0.59	0.73
−2.84	−3.69	0.22	0.85	
−4.95	−4.06	0.31	0.89	
4.87	4.41	0.29	0.46	
2.96	3.89	0.35	0.93	
1.65	1.02	0.25	0.63	
